# Targeting Neurogenesis in Seeking Novel Treatments for Ischemic Stroke

**DOI:** 10.3390/biomedicines11102773

**Published:** 2023-10-13

**Authors:** Takayuki Nagase, Kyohei Kin, Takao Yasuhara

**Affiliations:** Department of Neurological Surgery, Okayama University Graduate School of Medicine, Dentistry, and Pharmaceutical Sciences, Okayama 700-8558, Japan; me422075@s.okayama-u.ac.jp (T.N.); tyasu37@cc.okayama-u.ac.jp (T.Y.)

**Keywords:** neurogenesis, ischemic stroke, cell therapy, rehabilitations

## Abstract

The interruption of cerebral blood flow leads to ischemic cell death and results in ischemic stroke. Although ischemic stroke is one of the most important causes of long-term disability and mortality, limited treatments are available for functional recovery. Therefore, extensive research has been conducted to identify novel treatments. Neurogenesis is regarded as a fundamental mechanism of neural plasticity. Therefore, therapeutic strategies targeting neurogenesis are thought to be promising. Basic research has found that therapeutic intervention including cell therapy, rehabilitation, and pharmacotherapy increased neurogenesis and was accompanied by functional recovery after ischemic stroke. In this review, we consolidated the current knowledge of the relationship between neurogenesis and treatment for ischemic stroke. It revealed that many treatments for ischemic stroke, including clinical and preclinical ones, have enhanced brain repair and functional recovery post-stroke along with neurogenesis. However, the intricate mechanisms of neurogenesis and its impact on stroke recovery remain areas of extensive research, with numerous factors and pathways involved. Understanding neurogenesis will lead to more effective stroke treatments, benefiting not only stroke patients but also those with other neurological disorders. Further research is essential to bridge the gap between preclinical discoveries and clinical implementation.

## 1. Introduction

Ischemic stroke is one of the most important causes of long-term disability and death worldwide [1]. Numerous efforts have been made to reduce ischemia-induced neuronal injury and restore neurological function through various mechanisms [2]. Recombinant tissue plasminogen activator and mechanical thrombectomy are the only two therapeutic options for ischemic stroke approved by the United States Food and Drug Administration [3]. However, it must be used within 4.5 h of onset and is only available for a restricted patient population. Therefore, therapeutic treatments with a longer time window are needed.

Damaged neurons in conditions such as stroke were once believed to be incapable of regeneration as neurogenesis was believed to cease shortly after birth [3]. However, in 1998, adult neurogenesis in the human brain was discovered under physiological conditions [4]. This led to the notion that the proliferation of adult neural stem cells (NSCs) in the central nervous system may have the potential to replace lost or damaged neurons. The transplantation of exogenous stem cells and the stimulation of endogenous stem cells have emerged as potential therapeutic approaches for ischemic stroke and other neurodegenerative disorders. In the mammalian brain, neurogenesis occurs in at least two neurogenic niches, namely, the subventricular zone (SVZ) of the lateral ventricles and the subgranular zone (SGZ) of the dentate gyrus in the hippocampus, where intrinsic NSCs gradually undergo cellular proliferation, division, differentiation, migration, and integration to become functional neurons [5,6]. Neurogenesis is now widely accepted as a fundamental mechanism of neural plasticity [7]. Neurogenesis following ischemic stroke is recognized as a potential mechanism for neuronal recovery. However, endogenous neurogenesis alone is inadequate for effective brain repair, as the majority of newborn neurons do not survive [3].

**Figure 1:**
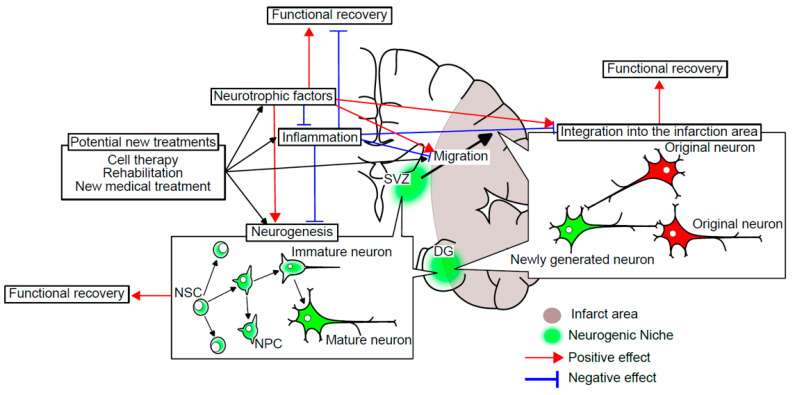
Current strategy for developing new treatments for ischemic stroke. Functional recovery from ischemic stroke was observed via a complicated pathway including neurogenesis, neurotrophic factors, and inflation. All of these factors have been focused on as targets of potential new treatments for ischemic stroke.

Facilitating regenerative repair processes such as neurogenesis and remodeling of dendritic spines holds promise as a novel therapeutic strategy for stroke treatment [8]. Therapeutic strategies involving cell therapy, rehabilitation, and pharmacotherapy-induced stimulation of endogenous neurogenesis represent potential approaches for repairing and regenerating the injured brain, offering a second therapeutic time window for ischemic stroke treatment (Figure 1) [9].

There has been extensive research discussing the association between neurogenesis and cerebral infarction. Despite this, a comprehensive understanding of knowledge remains lacking. In this review, we will summarize the knowledge of the relationship between neurogenesis and treatment for ischemic stroke so as to shed light on the current landscape and our current standpoint.

## 2. The Relationship between Neurogenesis and Ischemic Stroke

### 2.1. The Mechanism of Ischemic Cell Death

Ischemic stroke results in the deprivation or interruption of blood flow to the brain, leading to ischemic cell death in neuronal cells due to insufficient blood supply. Although the underlying mechanisms are not fully understood, our understanding of certain aspects has increased through years of research. When blood supply is disrupted, the maintenance of resting membrane potential, which is the most energy-consuming function of neuronal cells, is compromised. As a result, neurons undergo a process known as anoxic depolarization. Depolarized neurons release glutamate, which further depolarizes adjacent neurons and triggers additional glutamate release, creating a positive feedback cycle. Glutamate acts through N-methyl-D-aspartate receptors, inducing the influx of large amounts of intracellular calcium. Calcium also enters neurons through voltage-gated calcium channels during depolarization. Mitochondria serve as the primary source of calcium uptake in neurons and expand to buffer calcium, leading to mitochondrial dysfunction. Calcium-mediated protein degradation, lipid breakdown, and DNA fragmentation contribute to cellular necrosis [10,11]. Other molecular actions, such as the generation of free radicals in compromised mitochondria, also contribute to early neuronal cell death [12,13]. This process, known as excitotoxicity, was first reported in stroke in the late 1980s and served as a driving force for many stroke clinical trials in the 1990s [14]. However, limited advances were made with these trials [13]. This may mean that excitotoxicity is not a good target for treatment. Of course, we need to keep in mind that excitotoxicity involves complex mechanisms, including neuroinflammation, apoptosis, and reactive oxygen species metabolism, with numerous molecules playing roles that have yet to be fully identified [12]. Ongoing research in this field continues, and elucidating the detailed mechanisms will aid in the development of novel neuroprotective drugs.

### 2.2. Change in Neurogenesis after Cerebral Infarction

In the adult brain following ischemic stroke, it has been revealed that the production of neural progenitor cells significantly increases compared to the quiescent state (Figure 1) [15]. The generated neural progenitor cells migrate towards the damaged brain region, differentiate into mature neurons, establish appropriate long-distance connections, and potentially contribute to the recovery from ischemic stroke by integrating into the neural circuitry [16]. It has been reported that transplanted mesenchymal stem cells (MSCs) also migrate to these damaged brain regions, and this migration has been shown to be enhanced by electrical stimulation [17]

After cerebral ischemia, astrocytes become activated and through the Notch signaling pathway, they are capable of generating neurons in the adult mouse striatum in vivo [18]. Furthermore, it has been demonstrated that striatal astrocytes differentiate into immature neurons one week after middle cerebral artery occlusion (MCAO) and into mature neurons two weeks later [19]. Additionally, these astrocyte-derived neurons are able to form synapses with other neurons by the 13th week after MCAO, indicating their potential to establish connections with other neurons in the damaged brain [19]. 

Additionally, astrocytes have been implicated in adult neurogenesis by releasing neurotrophic factors [20]. In stroke models, activated astrocytes are found to enhance the expression of brain-derived neurotrophic factor (BDNF), promote the differentiation of neural precursor cells derived from the central nervous system, and improve the survival and engraftment rates of early NSCs [21]. Glial cell line-derived neurotrophic factor (GDNF), another neurotrophic factor secreted by astrocytes, has been shown to induce the neural differentiation of precursor cells and facilitate striatal neurogenesis after ischemic stroke in adult rats [22].Nerve growth factor (NGF), expressed by astrocytes, has been shown to be upregulated in the peri-infarct region after ischemic stroke and improve the survival of newly generated cells in the ipsilateral striatum and SVZ [23].

A major limitation of endogenous neurogenesis in the brain is the suggested lack of survival of high-quality newborn neurons [3,24]. After stroke, although a large number of new neurons are generated, over 80% of them die within the first two weeks, and the majority of them fail to differentiate into mature neurons beyond the fourth week post-stroke [25]. In rodents [26] and humans [27], stroke has been shown to promote neurogenesis in the SVZ and attract newborn neurons to the injury site, yet approximately 5% to 10% of newborn granule cells exhibit significant morphological abnormalities. 

In summary, ischemia-induced neurogenesis contributes to brain recovery to some extent, although its effects have been demonstrated to be modest. Endogenous neurogenesis alone is inadequate for effective post-stroke brain repair. To enhance post-stroke brain recovery efficiently, it is essential to adopt improved strategies, including augmenting the number of surviving neurons, ameliorating morphological abnormalities, diversifying cell subtypes, and constructing novel neural networks [3].

### 2.3. How Neurogenesis Contributes to Functional Recovery from Ischemic Stroke

The correlation between neurogenesis and the functional recovery in patients suffering from ischemic stroke is an area of active investigation. Following an ischemic stroke event, recently generated neurons migrate to the areas of the brain affected by damage, potentially contributing to functional recovery through their integration into neural networks, thereby reinstating lost or impaired neural pathways [28,29]. Furthermore, while the formation of new neurons has been suggested to potentially enhance functional recovery through its contribution to synaptic plasticity, conclusive evidence supporting this notion is lacking [30].

It has been demonstrated that neurogenesis is activated in the brain following stroke; however, endogenous neurogenesis alone is insufficient for brain repair. Therefore, methods that can more effectively activate neurogenesis and improve post-stroke symptoms through therapeutic interventions are being studied [24]. Cell therapy, exercise therapy as rehabilitation, and pharmacotherapy are expected to be such therapeutic interventions. The influences of the aforementioned neurotrophic factors and the post-stroke inflammation on neurogenesis have been reported [31]. These would be a good target. If the detailed molecular mechanisms governing neurogenesis activation following stroke are available, it would lead to new molecular approaches. However, it remains elusive [32], although the phosphatidylinositol-3-kinase (PI3K) pathway [33], Wnt/beta-catenin pathway [34], and Sonic Hedgehog pathway [35] were suggested to be implicated in neurogenesis activation.

As discussed later in this review, a significant amount of research has been conducted, and in general, these therapies have been shown to enhance neurogenesis and improve motor function. Further, some have reported the correlation between neurogenesis and treatment effect (Figure 1). Based on these findings, it is believed that the activation of neurogenesis after stroke can contribute to the improvement of post-stroke symptoms.

## 3. Neurogenesis in New Treatments for Ischemic Stroke

### 3.1. Cell Therapy

In recent years, a great many preclinical studies and clinical trials have been conducted on stem cell transplantation. Therapies using stem cells have shown promising results in preclinical trials, with observed reductions in infarct size and recovery of neural function [36]. Cell therapies appear to protect and repair damaged brain tissue after stroke. However, the underlying mechanism remains incompletely understood. The results of animal experiments utilizing cell therapy suggested that promoting neurogenesis may contribute to protecting and repairing damaged brain tissue after stroke. However, the detailed role of neurogenesis in the treatment effect has not yet been established. Other underlying mechanisms for the treatment effect have also been reported. Attenuation of neuroinflammation, secretion of neurotrophic factors, and augmentation of angiogenesis have also been reported to contribute to the treatment effect of cell therapy for ischemic stroke (Figure 1) [3]. In addition, they can also increase endogenous neurogenesis. Therefore, we need to keep in mind that the causal relationship between cell therapy-induced neurogenesis and the treatment effect of cell therapy is not well established.

#### 3.1.1. MSCs

MSCs are a heterogeneous population of pluripotent and multipotent cells found in the trabecular bone of nearly all bones, adipose tissue, umbilical cord, and dental pulp. Due to their ease of collection, ethical considerations, pluripotency, and ability to be safely transplanted, MSCs have been widely utilized in animal experiments and clinical trials for the treatment of ischemic stroke [17,37,38,39,40]. MSC transplantation has been reported to secrete growth factors, enhance expression of angiogenic factors and vascular density, reduce scar size, and limit apoptosis, thereby exerting beneficial effects on neurological recovery following ischemic brain injury in rats [41]. Additionally, while intracerebral transplantation is the primary delivery method, there have been reports of MSC transplantation via tail vein injection in rats, resulting in increased neurogenesis [42]. This may suggest the solid effect of MSCs on neurogenesis. 

MSC transplantation provides other beneficial effects. It has been reported to inhibit microglial activation and modulate immune response [41,43]. Such immunological response and inflammation were reported to affect endogenous neurogenesis [44,45]. Therefore, we must pay attention to these factors when evaluating the changes in neurogenesis induced by MSCs.

In recent years, genetically modified MSCs, such as vascular endothelial growth factor (VEGF)-transfected MSCs and SB623 cells which are human bone marrow stromal cells transfected with Notch1 intracellular domain, have been used for alternative approaches [40,46]. Such modification of MSCs can increase the effect on neurogenesis and functional recovery. It also aids in our understanding of the underlying mechanisms of MSC transplantation therapy.

#### 3.1.2. Neural Stem Cells (NSCs) and NPCs

NSCs can renew themselves and possess multipotency. The discovery of NSCs was a great milestone in the concept of neurogenesis [47]. NSCs can be identified in the SGZ in the dentate gyrus of the hippocampus and the SVZ of the lateral ventricles where endogenous neurogenesis can be identified [32,48]. After transplantation of NSCs from fetal neocortical tissue was reported to improve the behavioral outcome of ischemic stroke in animal experiments [49], a great deal of basic research, especially basic research with human fetal NSCs, has been conducted. NSCs were reported to differentiate into neuroblasts or neurons and to survive for a long time [50,51]. At the same time, endogenous neurogenesis was reported to be augmented by NSC transplantation [52]. Considering that several neurotrophic factors, including VEGF and BDNF, are secreted from NSCs, these are reasonable findings [52].

NPCs, which are more specialized and more considered the intermediate between stem cells and differentiated neurons, derived from adult brain and embryonic/fetal tissues have the capacity to differentiate into neurons, astrocytes, or oligodendrocytes [53]. As NSCs do not consistently differentiate into NPCs after transplantation, the direct implantation of NPCs can be a potentially superior choice for neuronal replacement [52,54]. When transplanted into the brains of individuals with cerebral infarction, NPC transplantation has been shown to increase the length and branching of dendrites and improve sensorimotor function [55]. These beneficial effects are believed to be associated with the secretion of neurotrophic factors such as BDNF, VEGF [56], GDNF [57], and basic fibroblast growth factor (bFGF) [58].

#### 3.1.3. Other Types of Cell Therapy

Cell therapy for ischemic stroke with other types of cells has also been reported. Induced pluripotent stem cells (iPSCs) have drawn the interest of scientists and are considered a promising source for the treatment of ischemic stroke. iPSCs are typically generated by reprogramming adult somatic cells, such as skin cells or blood cells. Several animal experiments revealed the beneficial effects of iPSCs on ischemic stroke, such as angiogenesis, modulated immune response, and anti-inflammatory effects [59,60]. Neurogenesis is also reported to be promoted by iPSCs [61]. However, the application of iPSCs did not always result in functional recovery. Optimizing the various methods, including the cultivation and differentiation of iPSCs and transplantation, is needed [59].

Differentiated cells from stem cells are also reported to be good candidates for cell therapy for ischemic stroke. Neural cells differentiated from dental pulp tissue, iPSCs, and human neural stem cells are examples of such differentiated cells. All of them are reported to contribute to neurogenesis augmentation and improve behavioral deficit due to ischemic stroke [62,63,64]. However, it must be noted that research with these cells is currently relatively limited and the reproducibility of experiments is a concern.

### 3.2. Rehabilitation

Rehabilitation has been regarded as one of the most important therapeutic interventions and solid strategies for functional recovery after ischemic stroke [65]. Mounting evidence from both clinical and basic research has shown that rehabilitation improved motor and cognitive function. In the clinical field, rehabilitation in inpatient rehabilitation facilities significantly increased motor function [66]. Virtual rehabilitation improved the motor and cognitive function of stroke patients [67]. Unfortunately, neurogenesis in humans has not yet been addressed. However, basic research showed that rehabilitation can facilitate neurogenesis as well as improve functional recovery. In rodent studies, enriched environments, forced running, or forced swimming (mimicking rehabilitation) are used as rehabilitative interventions. Similar to the clinical studies, rodent studies reported that motor and cognitive function were improved by enriching the environment with running wheels, tunnel systems, or climbing possibilities [68]. To identify the underlying mechanism of the treatment effect of rehabilitation, numerous animal studies have been conducted, some of which provide insights into neurogenesis. An enriched environment promoted the survival of newly born neurons [69,70] and resulted in the migration of neural progenitor cells to the peri-infarct region [71,72]. Housing rats in an enriched environment increases dendritic arborization in the cortex contralateral to the stroke lesion [68], enhances neurogenesis in the SVZ [73], and promotes axonal sprouting and crossover from the contralesional corticospinal tract to the denervated side of the spinal cord in stroke animals [74]. Another study showed that after cortical infarcts in the forelimb sensorimotor cortex, environmental enrichment as well as daily reaching training of the impaired paw both increased dentate neurogenesis and improved functional performance in the Morris water maze [75]. On the other hand, some studies suggested that neurogenesis may not contribute to the treatment effect of rehabilitation. For example, one rat study reported that running wheel training improved motor function after endothelin-1-induced stroke without affecting neurogenesis [76]. Considering the variety of start and end points of rehabilitation, such inconsistent results are not surprising. Further, various types of rehabilitation, namely, how enriching environments are, have been reported. Different rehabilitation strategies would definitely affect the outcome. A longitudinal study investigating the impact of the start and end points of rehabilitation or a comparison study of various types of rehabilitation would facilitate our understanding of the relationship between rehabilitation and neurogenesis. Rehabilitation also affects the immune system and facilitates the internal expression level of neurotrophic factors. As discussed above, these changes also contribute not only to the functional improvement but also to the augmentation of neurogenesis. Therefore, such cross talk should always be taken into account.

### 3.3. Pharmacotherapy

As discussed, limited treatments are available for the functional recovery from ischemic stroke. Therefore, various types of medical treatments have been assessed toward the development of new treatments for functional recovery. To the best of our knowledge, neurogenesis itself has not been focused on as a target of such medical treatment. However, several studies have reported medical treatments that increased endogenous neurogenesis. 

Several medical treatments for ischemic stroke or ischemic stroke-related diseases have been reported to modulate neurogenesis in basic research. HMG-CoA reductase inhibitors (statins) are widely used to lower cholesterol levels in vascular diseases and have been shown to be effective in reducing the risk of stroke and improving long-term outcomes [77]. Administration of atorvastatin has been reported to contribute to anatomical and functional recovery and to promote the neurogenesis and regeneration of noradrenergic neurons after cerebral ischemia [77]. Clinical trials have demonstrated the effectiveness of statins in reducing the risk of recurrent strokes and improving overall outcomes in individuals with a history of stroke, but there is no consensus on their routine use in the acute phase [78]. Edaravone is a drug used as a neuroprotective agent in the treatment of ischemic stroke. Its mechanism is believed to involve a reduction in reactive oxygen species generation after cerebral ischemia. It has also been reported that edaravone promotes neurogenesis [79]. Metformin, a widely used drug for the treatment of type 2 diabetes, is known to be an activator of adenosine monophosphate-activated protein kinase (AMPK) [80,81]. AMPK functions as an energy balance sensor and is rapidly activated in response to low energy supply [82]. AMPK is abundant in the brain, and its activation in the brain rapidly occurs in response to cerebral ischemia, which significantly increases angiogenesis and neurogenesis [83]. Chronic metformin treatment after stroke has been shown to improve functional recovery following ischemic stroke [80]. There were no clinical trials showing that metformin improved symptoms in post-stroke patients.

Basic research also revealed that medical treatments in other fields promote neurogenesis and accelerated functional recovery after ischemic stroke. Granulocyte colony-stimulating factor (G-CSF) is a hematopoietic growth factor that is used to treat and prevent neutropenia during cancer treatment. Intranasal administration of G-CSF showed a neuroprotective effect for a rat model of ischemic stroke and promoted neurogenesis [84]. In clinical trial, G-CSF was well tolerated in patients with acute ischemic stroke, but did not contribute to functional recovery and did not reduce infarct volume at 3 months after onset, compared with the control group [85]. Erythropoietin (EPO) is commonly used to treat anemia due to cancer or its treatment. It has been reported to promote cytoprotection and neurogenesis in rats after neonatal ischemic stroke [86]. EPO therapy significantly improved long-term neurological outcomes in patients after ischemic stroke in a clinical trial [87]. Fluoxetine, a selective serotonin reuptake inhibitor, has recently gained significant attention as a neuroprotective therapeutic agent. There is ample evidence in animal models of stroke demonstrating improved neurogenesis with fluoxetine treatment following cerebral ischemia [88]. In a clinical trial, treatment with fluoxetine for 90 days after ischemic stroke improved the long-term neural functional outcomes. Sildenafil, a phosphodiesterase type 5 inhibitor, increases cyclic guanosine monophosphate (cGMP) levels and is used for male sexual dysfunction. In basic research for ischemic stroke, sildenafil was reported to mediate multiple pathways and exert neurogenesis and significant neuroprotective effects on injured neurons in acute ischemic stroke [89,90]. Although the safety of sildenafil has been confirmed in clinical trials for the treatment of stroke, its effectiveness has not been verified [91]. All of these findings may be useful for other diseases in the clinical field. Ischemic stroke may be a new target in the near future following some clinical trials. 

Reagents that are not available as medicine in the clinical field are also reported to show neurogenesis augmentation and a neuroprotective effect for ischemic stroke. Angiopoietin-1 has emerged as an important regulator of physiological and pathological angiogenesis from the embryonic to postnatal stages. It has been reported to promote both functional recovery and neurogenesis in a rat model of focal cerebral ischemia [92]. P7C3-A20, which is a highly active neuroprotective aminopropyl carbazolea, has been reported to show beneficial effects for various types of central nervous system disorders in basic research. It also induced neurogenesis augmentation and functional improvement in a rat model of ischemic stroke [93,94]. Adiponectin is an adipocyte-derived secretory protein that plays various physiological roles in the nervous systems and can boost neurogenesis in the dentate gyrus of the hippocampus [95]. Neurogenesis in a rat model of ischemic stroke was also stimulated by adiponectin and promoted functional recovery [96]. In basic research, these reagents exhibit considerable promise as candidates for innovative medical interventions. Nevertheless, substantial work is required to make them available to the clinical field. Hopefully, the ongoing endeavors of researchers will deliver them to clinical patients in the near future.

### 3.4. Other Types of Interventions

Other types of interventions that aim to ameliorate the functional deficits of ischemic stroke are also reported to affect neurogenesis. Although many of them are not well recognized, they have potential to be innovative treatments for ischemic stroke.

Hyperbaric oxygen therapy (HBOT) has been found to promote neurogenesis and enhance cellular migration to the penumbra region. Additionally, HBOT increases the expression of BDNF, further facilitating the reconstruction of impaired neural networks and potentially contributing to the restoration of motor function. The involvement of the ROS/HIF-1α/β-catenin pathway in these processes has also been suggested [97]. Functional electrical stimulation has been reported to promote neurogenesis in the brains of acute ischemic stroke rats. It has been shown to enhance the expression of basic fibroblast growth factor and epidermal growth factor, ultimately improving motor function in rats [98]. Repetitive transcranial magnetic stimulation (rTMS) has been widely used in clinical trials as a post-stroke rehabilitation method in ischemic stroke and has been shown to alleviate post-stroke functional impairments. High-frequency rTMS is reported to promote neurogenesis and improve functional recovery by activating the BDNF/TrkB signaling pathway [99]. Additionally, it has been demonstrated that combining rTMS with human NSC transplantation can enhance functional recovery following ischemic stroke in rats. This combination not only significantly increased neurogenesis and protein levels of BDNF, but rTMS also facilitated the neuronal differentiation of hNSCs [100]. Constraint-induced movement therapy (CIMT) is an effective approach for improving functional recovery following ischemic stroke. The neuroprotective and functional recovery effects of CIMT have been reported to be mediated by increased endogenous HIF-1α and VEGF expression, followed by subsequent neurogenesis and angiogenesis [101].

## 4. Potential and Prospects of Neurogenesis in Treatment for Ischemic Stroke

As discussed above, various methods, including cell therapy, rehabilitation, and pharmacotherapy, have been reported to activate neurogenesis. It has also been demonstrated that there is a correlation between the activation of neurogenesis and the extent of improvement in motor, cognitive, and other aspects of function. These findings suggest that neurogenesis may be a promising target for developing new treatments for ischemic stroke. However, we must keep in mind that there is not yet solid evidence demonstrating that an increase in neurogenesis contributes to functional recovery in ischemic stroke. We understand that addressing this question is challenging. Nevertheless, it is imperative to confront the importance of this question. Utilizing transgenic mice such as Nestin-δ-HSV-TK-EGFP transgenic mice, which exhibit reduced neurogenesis, may be a powerful tool to address this question [102]. Precisely designed radiation for the SVZ and SGZ stands out as another potent method.

Although some pathways and factors involved in the mechanism of neurogenesis have gradually been elucidated, there are many aspects that remain unknown. As our understanding of the mechanisms and associated molecules of neurogenesis continues to advance, it is expected that more effective approaches will be identified. Progress will be beneficial for both ischemic stroke and various neurological disorders where therapeutic advancements remain insufficient.

## 5. Conclusions

In basic research, extensive treatment successfully achieved functional recovery for ischemic stroke and increased neurogenesis was observed. Regardless of the types of treatment, such as cell therapy, rehabilitation, and pharmacotherapy, neurogenesis augmentation was observed. Further, considering that neurogenesis itself would contribute to functional recovery in nature, neurogenesis can contribute to functional recovery by such treatment. Nonetheless, despite the exploration of several therapeutic approaches, including cell therapy, rehabilitation, and pharmacotherapy, only a limited number have progressed to clinical implementation. A poor understanding of neurogenesis may be one of the reasons. Therefore, further research is strongly needed to gain a deeper understanding of the intricate mechanisms governing neurogenesis in the context of stroke. Such advancements are essential for the development of more efficacious and targeted therapeutic strategies that can be translated into clinical treatment for ischemic stroke.

## Data Availability

Not applicable.
